# Testing the Efficacy of OurSpace, a Brief, Group Dynamics-Based Physical Activity Intervention: A Randomized Controlled Trial

**DOI:** 10.2196/jmir.5342

**Published:** 2016-05-06

**Authors:** Brandon Irwin, Daniel Kurz, Patrice Chalin, Nicholas Thompson

**Affiliations:** ^1^ Digital Physical Activity Laboratory Department of Kinesiology Kansas State University Manhattan, KS United States; ^2^ Dependable Software Research Group Santa Cruz, CA United States

**Keywords:** physical activity, group dynamics, social media, cohesion, Internet, social support

## Abstract

**Background:**

Emerging technologies (ie, mobile phones, Internet) may be effective tools for promoting physical activity (PA). However, few interventions have provided effective means to enhance social support through these platforms. Face-to-face programs that use group dynamics-based principles of behavior change have been shown to be highly effective in enhancing social support through promoting group cohesion and PA, but to date, no studies have examined their effects in Web-based programs.

**Objective:**

The aim was to explore proof of concept and test the efficacy of a brief, online group dynamics-based intervention on PA in a controlled experiment. We expected that the impact of the intervention on PA would be moderated by perceptions of cohesion and the partner’s degree of presence in the online media.

**Methods:**

Participants (n=135) were randomized into same-sex dyads and randomly assigned to one of four experimental conditions: standard social support (standard), group dynamics-based–high presence, group dynamics-based–low presence, or individual control. Participants performed two sets of planking exercises (pre-post). Between sets, participants in partnered conditions interacted with a virtual partner using either a standard social support app or a group dynamics-based app (group dynamics-based–low presence and group dynamics-based–high presence), the latter of which they participated in a series of online team-building exercises. Individual participants were given an equivalent rest period between sets. To increase presence during the second set, participants in the group dynamics-based–high presence group saw a live video stream of their partner exercising. Perceptions of cohesion were measured using a modified PA Group Environment Questionnaire. Physical activity was calculated as the time persisted during set 2 after controlling for persistence in set 1.

**Results:**

Perceptions of cohesion were higher in the group dynamics-based–low presence (overall mean 5.81, SD 1.04) condition compared to the standard (overall mean 5.04, SD 0.81) conditions (*P*=.006), but did not differ between group dynamics-based–low presence and group dynamics-based–high presence (overall mean 5.42, SD 1.07) conditions (*P*=.25). Physical activity was higher in the high presence condition (mean 64.48, SD 20.19, *P*=.01) than all other conditions (mean 53.3, SD 17.35).

**Conclusions:**

A brief, online group dynamics-based intervention may be an effective method of improving group cohesion in virtual PA groups. However, it may be insufficient on its own to improve PA.

## Introduction

Despite the broad health benefits of physical activity (PA) [1], only approximately 5% of US citizens are actually meeting PA recommendations [2]. The US Centers for Disease Control and Prevention have identified and advocate several evidence-based approaches to promoting PA, including social support-based interventions [3]. Among social support-based interventions are those that involve peer groups [4] and other group-based approaches [5]. Meta-analysis data show that highly effective group interventions are those that include group dynamics-based activities [5]. Group dynamics-based interventions include team-building activities (eg, group goal setting) to facilitate group member interactions with the ultimate goal of enhancing group cohesion [6]. In comparison to delivering interventions to collections of people and individual-based PA programs, group dynamics-based programs have been associated with higher levels of moderate-to-vigorous physical activity (MVPA), program adherence, and levels of social interaction [5]. However, there exist several drawbacks to the group dynamics-based approach to PA promotion—groups are often required to meet in person, which may restrict participation due to geographic location and requires participants to coordinate meeting times amid busy schedules. Additionally, there is a burden placed on staff and practitioners to manage and facilitate the group activities. Thus, strategies that overcome these challenges may help optimize group dynamics-based interventions and free resources to allow for broader reach and effectiveness.

One such strategy might involve the use of the Internet and, in particular, virtual teams [7]. The Internet provides a unique potential for a vast population of people, both sedentary and active, to seek out health information and/or support for behavior change. Additionally, Internet-based tools (eg, social media) can and should facilitate group interactions [8,9], thus decreasing the burden of staff/practitioners. This potential has not gone unnoticed because Internet-based interventions are now being used more often for promoting positive health behaviors, such as PA [10-12]. However, although significant, the overall effects of Internet-delivered interventions focusing on PA promotion have been small [13] and are prone to a variety of drawbacks. For example, participant attrition in Internet-based weight loss programs is typically high (>25%) and those who adhere to the programs often have reduced engagement over time [14]. Given the effectiveness of group dynamics-based programs to impact MVPA and enhance program adherence as well as the ability of the Internet to overcome traditional barriers associated with face-to-face interventions, a sensible strategy for improving Internet-based interventions would be to translate group dynamics-based practices into an Internet-based intervention.

In this study, we developed a brief, online group dynamics-based PA intervention to lead users through a series of virtual team-building activities. The app was designed according to Carron and Spink’s [15] team-building model and targeted several key aspects of cohesion development, including group environment, group structure, and group processes. Because many existing Internet-based interventions include peer social support components, we compared the effect of a group dynamics-based intervention with that of a common social support app (ie, a moderated discussion board). We also wished to test for the effect of the intervention on known correlates and mediators of PA, including enjoyment [16,17], perceived exertion [18], and self-determined motivation [19,20].

The primary aim of this study was to test the efficacy of a brief, online group dynamics-based intervention in increasing PA. The secondary aims were to test the moderating effects of group cohesion and the presence of a partner. We created and randomized participants into one of four conditions (individual, standard, group dynamics-based–low presence, group dynamics-based–high presence) to test the following hypotheses:

1. Participants in a brief, group dynamics-based online intervention (group dynamics-based–high presence, group dynamics-based–low presence) will be more physically active than those in a standard social support intervention (standard) after controlling for baseline PA.
2. The impact of the intervention on PA will be moderated by perceptions of group cohesion (group dynamics-based>standard).
3. The impact of the intervention on PA would be moderated by the partners’ degree of presence (group dynamics-based–high presence>group dynamics-based–low presence).

## Methods

### Recruitment

Participants (N=135; 66 males, 69 females; mean age 19.54, SD 1.81 years) were recruited from an introductory level kinesiology course at a large Midwestern university to participate in a single session of a 1-hour “video game” study. Data were collected between March and December 2014. All participants were screened for health risks using the PA Readiness Questionnaire [21] and were awarded course credit for the completion of the study. An alternate assignment for credit was available for the students who did not participate in the research study. Ethical approval for the study was granted by the University’s Institutional Review Board (IRB #6318.1).

### Design

Similar to previous studies testing the impact of group dynamics on PA [22-24], we used a brief intervention in a laboratory setting to test our hypotheses. There are a number of advantages to this approach, including control of extraneous variables and the ability to test the efficacy of the core features of an intervention prototype at minimal financial and human cost. The present study used a randomized 2 (gender) × 4 (condition) × 2 (block) experimental design with repeated measures on the last factor. Each block consisted of an identical series of five planking exercises: front plank, side plank (left), one-leg plank (left), side plank (right), and one-leg plank (right).

**Figure 1:**
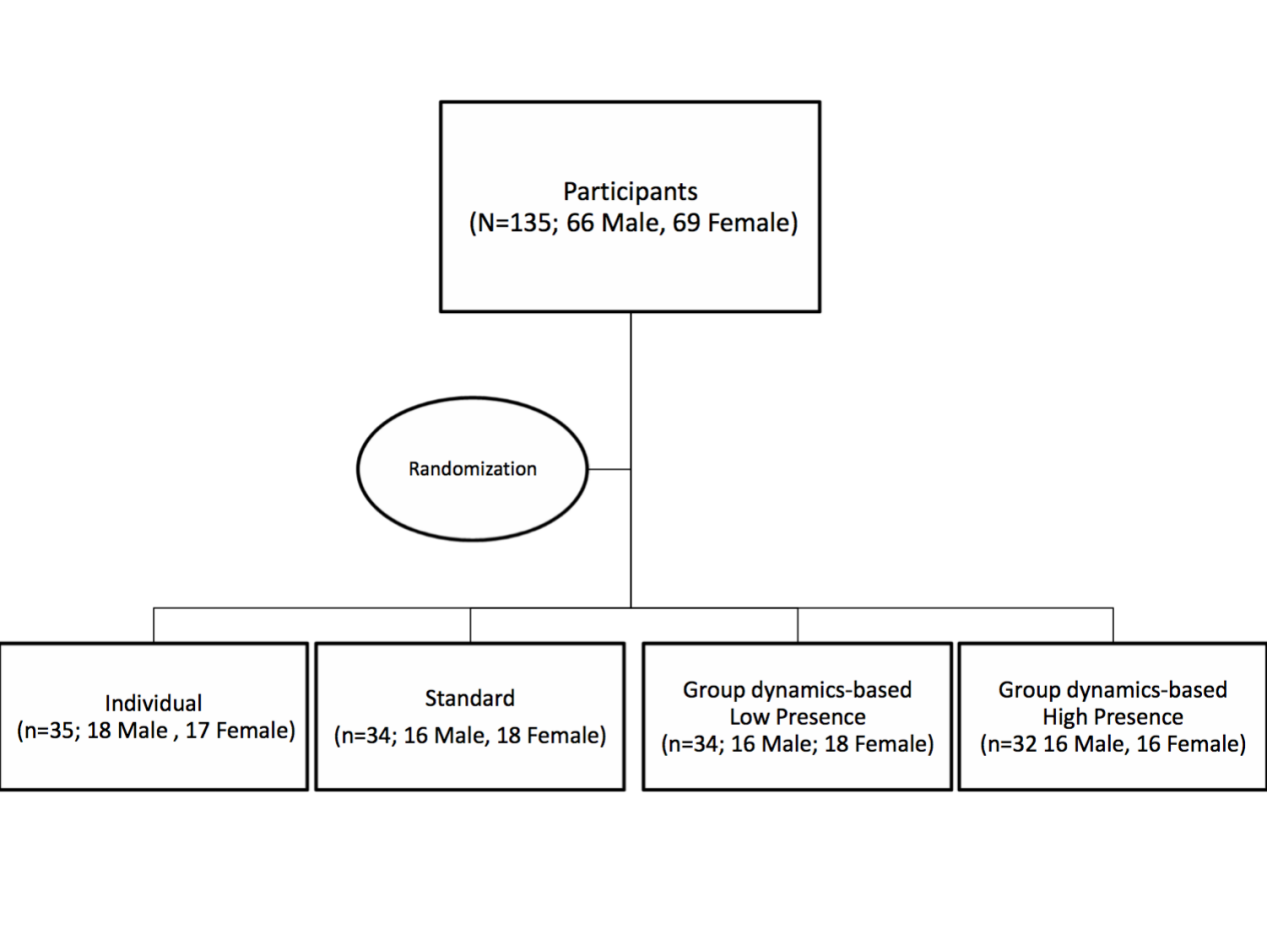
Flowchart of participant distribution.

Participants were randomly selected from a participant pool and asked to provide times and dates they were available to participate. Participants were then, unknowingly, assigned to same-sex dyads based on their availability. Dyad members were scheduled to participate in the study concurrently. Individual dyad members were sent to separate testing rooms to avoid any interactions outside of the experiment. Three individuals were unable to be scheduled into a dyad; their results were included in the individual condition. On arrival to the laboratory, dyads were randomly assigned to a condition: individual (n=32, 16 dyads), standard social support (n=34, 17 dyads), group dynamics-based–low presence (n=34, 17 dyads), or group dynamics-based–high presence (n=32, 16 dyads) (Figure 1). In the case that a dyad member failed to appear for their session, the present member was told by the experimenter that “we are experiencing technical difficulties and unable to run the trial today” and was rescheduled for a future time slot (n=17; 14 males, 3 females).

### Procedures

Participants arrived individually, signed an informed consent form, and were instructed to sit in an isolated room—separated from the experimenter and their partner—in front of a computer and began watching a video tutorial that included instructions for their participation during the experiment. Participants were given instructions for proper technique for a series of abdominal planking exercises that they would be performing during the experiment. A computer-generated trainer demonstrated the exercises during both the instructional video and during each block of exercise. Participants were instructed to hold each planking exercise for as long as they could without causing any undue discomfort or pain to themselves.

To minimize the risk of partners becoming aware of the other’s proximity, all participants were instructed to wear a pair of noise-canceling headphones for the full duration of the experiment. The headphones doubled as speakers for the computers. Participants were further instructed that if they needed assistance or had questions they should use a chat box provided on their computer, which directly linked them to the experimenter, in lieu of trying to verbally communicate.

Once participants completed the video tutorial, they were instructed to sit on an exercise mat, wait for the virtual trainer to start the exercise, and follow along with that trainer during each exercise (block 1). Once both dyad members were ready to begin, the experimenter initiated the virtual trainer and participants completed the first series of exercises independently and unaware of their partner. All participants performed the planks in the same order with a short (40 sec) rest period between each plank. During each planking exercise, participants were shown a live stream video of themselves exercising, allowing them to check their form against the virtual trainer. This constituted the first block of exercises (block 1).

At the end of block 1, all participants were asked to return to their computer and wait for further instructions. For participants in the individual condition, participants were given a 15-minute rest period in which they were told the average duration they held the planking exercises for, were asked to fill out a brief self-efficacy survey, and given generic reading material to occupy their time until block 2 began. In any of the partner conditions (standard, group dynamics-based–low presence, or group dynamics-based–high presence), participants were introduced to their partner through the Web app. Participants in a group dynamics-based condition received the full group dynamics-based app, whereas participants in the standard condition received a modified version of the app that removed the majority of team-building activities found in the full version. The standard version of the app was intended to mimic the features found in standard social support apps (eg, a discussion board), where communication is limited to text chat and minimally facilitated (eg, through prompts). In both versions, participants were given the following team task: “The two of you will be performing together as a team. Your team’s task is to hold the exercise for as long as possible. Your team’s time will be the total number of seconds that your team holds the exercises.” Block 2 began following the completion of the group dynamics-based app (when applicable) and a brief rest period.

For block 2, the individual condition followed the same procedures as block 1 whereas the group dynamics-based–low presence and standard conditions followed the same procedures as block 1 except they were now aware they had a partner and were given the aforementioned team task. Participants in the group dynamics-based–high presence condition followed the same procedure as the group dynamics-based–low presence and standard conditions; however, instead of seeing the live video stream of themselves exercising, they were shown the live stream of their partner instead (video streams were blurred to protect participants’ confidentiality). This set of planking exercises constituted the second and final block of exercises (block 2).

Following block 2, all participants returned to their computers to complete the final intrinsic motivation survey. Participants in partnered conditions also completed the cohesion questionnaire. Once completed, participants were thanked, debriefed, asked to not discuss the study with their classmates, and dismissed separately to avoid partners meeting each other in person.

### Group Dynamics-Based Intervention Description

The group dynamics-based intervention was a theory-based Web app (“OurSpace”) informed by Carron and Spink’s [15] team-building model. The app included several team-building activities to target different aspects of group dynamics, all of which are evidence-based practices for promoting group cohesion. We developed two versions of the app: a full version that included all group dynamics-based activities and a modified version that included only two activities (intended to mimic traditional social support tools commonly found in Internet-based interventions). In the full version of the app, participants entered their personal information, including first name (although their partner would see a pseudonym), gender, year in school, career goals, and something interesting about themselves, and selected an avatar from a list of generic preset characters. On the following page, each participant was asked to share something they struggled with during the exercises. Partners then exchanged advice on how the other could overcome their struggles (social support). Next, group distinctiveness was established by having the partners vote on and select a team icon and team name. Next, partners worked to solve a simple team-based puzzle together. Completion of the puzzle required partners to cooperatively control an onscreen character using directional arrows. One dyad member was given control of the character up/down movements, whereas the other controlled the right/left movement; coordination and cooperation were required to complete the task. Partners then established a group norm of what they believe the group’s expected effort level should be, individually and collectively, agreeing on the expected group effort value using a 1-10 scale. Finally, individual positions within the group were established by telling each dyad member how long they held each exercise and how long their partner held each exercise during block 1. The modified version of the app concluded after the social support page (app descriptions can be found in Table 1). The full version of the app was used by both group dynamics-based conditions, the modified version was used by the standard condition, and individuals did not use the app. Following the completion of the online session, participants reported the task-predicted performance survey and waited to begin the block 2 exercises.

**Table 1:** Description of the OurSpace app.

Theoretical construct and app feature		Group dynamics-based app	Standard app
**Group environment**			
	Share personal information	Yes	Yes
	Team name and icon	Yes	No
**Group structure**			
	Establish group exercise norms	Yes	No
	Establish positions within group	Yes	No
**Group process**			
	Team-based puzzle	Yes	No
**Social support**			
	Prompts to provide and receive support	Yes	Yes

### Measures/Outcomes

#### Physical Activity

Physical activity was operationally defined as the amount of time (in seconds) that participants persisted during block 2 after controlling for individual differences in ability and PA during block 1. The summed mean of the time spent performing the five planking exercises constituted a block score. Digital stopwatches were used to measure time spent in each exercise. Time was measured from the moment participants got into position for the exercise until the participant quit the exercise. The rest time between exercises was also calculated. This process was repeated for each plank exercise.

#### Perceptions of Cohesion

Participant’s perception of cohesion was measured using a modified Physical Activity Group Environment Questionnaire (PAGEQ) [25]. Original PAGEQ questions were modified to fit the context of the present study (eg, PAGEQ: “members of our PA group often socialize during exercise time” was modified to “members of our exercise group often socialized during time spent online”). Three items from the original PAGEQ items were omitted from the modified version due to lack of relevance within this study.

The modified PAGEQ measured participants’ perceived cohesion based on four dimensions: (1) attraction to group-task (ie, “I like the exercise done in this group”), (2) attraction to group-social (ie, “I enjoyed my social interactions within this online exercise group”), (3) group integration-task (ie, “our group is united in its beliefs about the benefits of the exercises offered in this program”), and (4) group integration-social (“members of our group would likely spend time together after the program ends”). Consistent with the original PAGEQ, each question was answered using a 9-point Likert scale, (ie, 1=very strongly disagree, 5=neither agree nor disagree, and 9=very strongly agree) [18]. Cronbach alpha was used to determine internal consistency reliability; scores for attraction to group-task (Cronbach alpha=.74), attraction to group-social (Cronbach alpha=.85), group integration-task (Cronbach alpha=.80), and group integration-social (Cronbach alpha=.76) were deemed acceptable.

#### Rating of Perceived Exertion

Ratings of perceived exertion (RPE) were measured using a 10-point RPE scale [26]. Scale measures ranged from 1 meaning “no exertion at all” to 10 meaning “maximal exertion.” Participants recorded their own RPEs on a sheet provided to them during the rest period immediately after completing each planking exercise. Scores were calculated as the mean reported RPE for each block.

#### Task Enjoyment

Task enjoyment was measured using a short 8-item (Cronbach alpha=.74) version of the PA Enjoyment Scale (PAES) [27,28]. Each item was rated on a 7-point bipolar scale beginning with the stem “Please rate how you feel at the moment about the PA you have been doing according to the following scales” (eg, 1=I loved it; 7=I hated it). Previous studies have shown high correlations with the longer 18-item scale (*r*=.94) [29] and strong reliability (Cronbach alpha=.91) [30].

#### Motivation

Motivation was measured with the Situational Motivation Scale (SIMS) [31]. The SIMS contained 16 items, which reflected different reasons a participant might be motivated to participate in the exercises. Each item was rated on a 7-point bipolar scale beginning with the stem “Please indicate the answer that best describes the reason why you are currently engaged in the abdominal exercises you are performing. Answer each item according to the following scale” (eg, 1=corresponds not at all; 7=corresponds exactly). There were four subscales (4 items each) based on Self-Determination Theory: amotivation (Cronbach alpha=.94), external regulation (Cronbach alpha=.95), identified regulation (Cronbach alpha=.95), and intrinsic motivation (Cronbach alpha=.93).

### Sample Power

An a priori power analysis following *F* index recommendations indicated that a sample size of n *=* 32 per condition would be sufficient for detecting a moderate (*F*=0 *.* 25) effect with probability >.80. Effect size was determined by a power analysis based on the findings of similar studies [22,23] using G-power software.

### Statistical Analyses

#### Hypothesis Testing

To test the hypotheses that (1) participants using a group dynamics-based app would have higher PA rates than those using the standard app and that (2) PA rates of participants using a group dynamics-based app would be moderated by levels of presence, a 4 (condition) × 2 (gender) ANCOVA was run with block 1 PA, baseline self-efficacy, and measures of intrinsic motivation as the covariates with block 2 PA as the dependent variable. To test the hypothesis that cohesion moderates the impact of PA rates a 3 (condition: all standard, group dynamics-based–low presence, group dynamics-based–high presence) × 2 (gender) MANOVA with each subscale of the modified PAGEQ as dependent variables.

#### Ancillary Analyses

The RPE (measured at the end of blocks 1 and 2) was analyzed with a 2-way condition × gender ANCOVA with block 1 RPE used as a covariate. Enjoyment (measured after block 2) was analyzed with a 2-way condition × gender ANOVA. Self-efficacy and all motivation subscales (measured at baseline, after block 1, and after block 2) were analyzed with separate 2-way condition × gender repeated measures ANCOVAs with baseline measures used as covariates. All analyses were conducted using SPSS 22 statistical software.

**Table 3:** Group cohesion by condition.

Cohesion variable and condition vs comparison condition			n	Mean (SD)^a^	*P*
**Attraction to group-task **					
	**Standard**		34	5.71 (1.37)	
		Group dynamics-based–low presence			.08
		Group dynamics-based–high presence			.39
	**Group dynamics-based–low presence**		34	6.40 (1.08)	
		Standard			.08
		Group dynamics-based–high presence			.68
	**Group dynamics-based–high presence**		32	6.13 (1.21)	
		Standard			.39
		Group dynamics-based–low presence			.68
**Attraction to group-social**					
	**Standard**		34	4.88 (1.19)	
		Group dynamics-based–low presence			.001
		Group dynamics-based–high presence			.17
	**Group dynamics-based–low presence**		34	6.06 (1.28)	
		Standard			.001
		Group dynamics-based–high presence			.19
	**Group dynamics-based–high presence**		32	5.48 (1.33)	
		Standard			.17
		Group dynamics-based–low presence			.19
**Group integration-task **					
	**Standard**		34	4.12 (1.18)	
		Group dynamics-based–low presence			<.001
		Group dynamics-based–high presence			.02
	**Group dynamics-based–low presence**		34	5.20 (1.04)	
		Standard			<.001
		Group dynamics-based–high presence			.43
	**Group dynamics-based–high presence**		32	4.86 (0.93)	
		Standard			.02
		Group dynamics-based–low presence			.43
**Group integration-social **					
	**Standard**		34	4.61 (1.64)	
		Group dynamics-based–low presence			.04
		Group dynamics-based–high presence			.48
	**Group dynamics-based–low presence**		34	5.46 (1.22)	
		Standard			.04
		Group dynamics-based–high presence			.44
	**Group dynamics-based–high presence**		32	5.02 (1.17)	
		Standard			.48
		Group dynamics-based–low presence			.44

^a^Perceived cohesion scales ranged from 1 to 9.

## Results

### Sample Population

The total sample consisted of 135 college-aged participants (66 males, 69 females; age mean 19.54, SD 1.81). No participants dropped out of the study before completing their sessions.

### Preliminary Analyses

An intraclass correlation analysis was run to detect potential agreement or “clustering” of PA and cohesion scores within dyads. Results for perception of cohesion were analyzed according Carron and colleagues’ [32] recommendations on determining the degree to which perceptions were shared within groups (or “groupness”). Criteria for detecting a small groupness effect was set at an intraclass correlation coefficient (ICC) of greater than or equal to .40 for attraction to group-social and attraction to group-task and an ICC of greater than or equal to .60 for group integration-social and group integration-task. Under these criteria, there was no evidence of a group clustering for perception of cohesion scores (attraction to group-task: ICC=.258, *P*=.15; attraction to group-social: ICC=.088, *P*=.37; group integration-social: ICC=.505, *P*=.008; group integration-task: ICC=.253, *P*=.16). Results of PA indicated that scores were not clustered in dyads (ICC=.173, *P*=.08) for any conditions (individual: ICC=.268 *P*=.15; standard: ICC=.067, *P=*.40; group dynamics-based–low presence: ICC=–.088, *P*=.64; individual: ICC=.341, *P*=.09). Thus, all following analyses of PA and perceived cohesion were conducted at the individual level.

### Physical Activity

Physical activity was significantly greater in the group dynamics-based–high presence condition than all other conditions, (*F*_3,121_ *=* 3.75, *P=*.01). There were no other significant differences between conditions. There was also a gender main effect; males were more physically active than females (*F*_1,121_=7.78, *P*=.006). Means of the analysis can be found in Table 2.

**Table 2:** Physical activity measured as mean persistence by condition and gender.

Condition	Overall			Male^a^		Female^a^	
	n	Mean (SD)	Range	n	Mean (SD)	n	Mean (SD)
Individual	35	50.56 (18.43)	15.6-87.2	18	55.74 (20.32)	17	45.07 (14.85)
Standard	34	54.98 (17.12)	27.4-86.0	16	57.18 (15.32)	18	53.03 (18.80)
Group dynamics-based–low presence	34	54.36 (16.50)	22.0-110.8	16	60.48 (16.98)	18	48.93 (14.41)
Group dynamics-based–high presence	32	64.48 (20.19)^a^	32.8-102.6	16	73.86 (17.07)	16	55.09 (19.05)

^a^Significant at the *P*<.05 level.

### Group Cohesion

There was significant main effect for condition (*F*_8,184_=2.77, *P*=.01). There were significant differences between conditions in all dimensions of cohesion (*P*<.001 to *P*=.04), except for attraction to group-task, (*P=*.06). Overall, where differences existed, mean scores were higher in the group dynamics-based conditions than the standard conditions. Specific differences were identified using a Scheffe post hoc analysis and are identified along with the means and standard deviations in Table 3.

### Ancillary Analyses

There were no significant differences in RPE between conditions (*F*_3,127_=1.83, *P*=.14) or gender (F_1,127_=0.30, *P*=.58), on enjoyment between conditions (*F*_3,127_=1.60, *P=*.19) or gender (*F*_1,127_=0.22, *P=*.64), or in motivation between conditions (intrinsic motivation: *F*_3,123_=0.50, *P*=.01; identified regulation: *F*_3,126_=0.41, *P*=.74; external regulation: *F*_3,126_=0.49, *P*=.68; amotivation: *F*_3,126_= 2.56, *P*=.06) or gender (intrinsic motivation: *F*_1,123_=0.03, *P*<.001; identified regulation: *F*_1,126_=0.68, *P*=.41; external regulation: *F*_1,126_=0.50, *P*=.48; amotivation: *F*_1,126_=0.13, *P*=.72) over time.

## Discussion

The primary aims of this study were to test the efficacy of a brief, online group dynamics-based intervention to increase PA. We also examined the ability of the intervention to impact group cohesion and tested the moderating effect of presence. We hypothesized that the group dynamics-based intervention would impact group cohesion and that higher perceptions of cohesion would be related to higher levels of PA. We also hypothesized that participants who were more visually present to their partner would be more physically active. Our hypotheses were partially supported. Although groups were more physically active than individuals; overall, the only intervention feature that impacted PA was the degree of the partner’s presence. Individuals were more active when their partner was virtually present during exercise. Although the intervention impacted the group cohesion mediating process, group cohesion was unrelated to PA. Potential explanations and implications for research and practice are discussed.

### Principal Results

#### Physical Activity

The hypothesis that the brief, online group dynamics-based intervention would impact PA was partially supported. Participants receiving the group dynamics-based intervention were more physically active than those exercising alone and those receiving a minimal social support intervention, but only when one’s partner was highly present. Further, although participants were more active in the high presence condition, our RPE data show that they did not perceive themselves to be working any harder than those in the less physically active conditions, suggesting that exercising with a highly present virtual partner may help overcome barriers related to exercise intensity [33]. Further, all measures of motivation were equal across conditions, suggesting that exercising with a partner under these conditions poses little risk for undermining one’s self-determined motivation for PA. This is an encouraging result because self-determined motivation is correlated with long-term maintenance of PA behavior change [20].

Our finding that the intervention did not impact PA even though we impacted the group cohesion mediator contradicts a large body of evidence linking group dynamics-based interventions, group cohesion, and PA [5,34]. However, there are a number of notable differences between the conditions within our brief, online group dynamics-based intervention for PA and previous group dynamics-based interventions for PA, including mode of delivery, dose of intervention, and the type of behavior targeted. First, our intervention was delivered entirely online, whereas previous group dynamics-based interventions have been delivered either face-to-face or partially online [35]. A completely online intervention has the obvious drawback of limiting the amount of communication, presence, and identifiability between participants, all of which are key factors impacting group dynamics and subsequent performance and PA. Despite our efforts in this intervention to enhance such group processes through the Web-based app, they may have failed to meet a minimum threshold for behavior change and may rely on face-to-face interactions to impact PA.

Second, the current intervention targeted PA intensity and duration, whereas many other group dynamics-based interventions target frequency of PA and program adherence. Participants in previous face-to-face group dynamics-based interventions for PA may be more physically active as a result of the intervention because of the intervention impact on frequency of PA bouts and/or attendance at program-related activities (ie, adherence) [5], not because they are more likely to exercise longer or harder within bouts than nonadherers. To date, research has not examined this claim. Third, the importance of the goal given to participants may have undermined their motivation to be physically active. Self-selected goals have been shown to lead to higher goal attainment and effort than non-self-selected goals [33]. In our intervention, groups were assigned the goal of holding the plank exercises for as long as they could. In previous group dynamics-based interventions, groups typically have the ability to select their own PA goals [35].

#### Group Cohesion

The second hypothesis that groups using the group dynamics-based app would report higher perceptions of cohesion compared to the standard social support app was supported. Results showed that, with the exception of attraction to group-task, perceptions of cohesion were higher for those who participated in the enhanced group dynamics-based intervention compared to those in the comparison condition who only received a minimal dose of social support. This finding is consistent with past face-to-face group dynamics-based studies in which the use of group dynamics-based principles has been shown to improve perceptions of cohesion among groups [6]. This finding is encouraging for online interventions with social support components, suggesting that such components might be fruitfully designed to impact group processes known to mediate the impact of interventions on PA. Further, our data suggest that impacting this mediator can be done rather swiftly, considering the study consisted of a single 1-hour visit during which only 7 to 9 minutes were spent using the group dynamics-based app.

#### Partner Presence

The third hypothesis, that increased group member presence would moderate PA, was supported. In fact, presence was the only factor to impact PA, in addition to the provision of performance feedback. This corroborates a large body of research showing the impact of virtual presence on group performance, including PA [36,37], but is the first to demonstrate these effects within the context of an online group dynamics-based intervention for PA.

### Implications

Our data suggest that impacting cohesion in online groups is feasible using a group dynamics-based app. More generally, this study suggests that online peer-to-peer interactions can be facilitated to impact group-level mediators of PA through purposeful, theory-based design. Given the obstacles and resources needed to implement and participate in face-to-face group-based interventions, further research and optimization of online group dynamics-based interventions such as this is warranted. As mentioned, future research can and should test the impact of an online group dynamics-based intervention on PA over longer durations and bouts of exercise (ie, adherence) and vary and test the dose of online versus face-to-face interaction on PA outcomes.

### Limitations

This study has several limitations. First, without a standard app high-presence condition we cannot conclude with certainty whether the higher levels of PA are attributable to the group dynamics-based components, the heightened presence of one’s partner, or a synergistic effect of both. Indeed, higher levels of online presence are associated with a variety of positive outcomes (eg, increased productivity, fewer antisocial behaviors, increased PA) and may be operating independently of group cohesion. This is likely the case because presence was manipulated only after participants had completed the team-building activities, not during them, and that all dimensions of cohesion were slightly higher in the group dynamics-based low-presence condition than in the high-presence condition, although these differences were nonsignificant. Future research would do well to untangle the impact of presence in online PA interventions and whether group dynamics-based components add value to such interventions. Second, we used a convenience sample of college students recruited from an introductory level kinesiology course, who may be more used to and respond differently to digital technology than other populations (eg, adults, elderly). Further, this was a brief intervention conducted in a laboratory with only one bout of exercise. Whether the impact of this intervention on cohesion and PA can be sustained over time and across different real-world settings warrants further study.

### Conclusions

This study tested an online group dynamics-based intervention to increase PA. Our brief intervention successfully impacted the mediating process, group cohesion, but did not impact PA. Additionally, virtual presence of group members had no effect on either perceptions of cohesion or PA.

In summary, online group dynamics-based apps may be a practical resource that can be used to overcome traditional barriers to utilizing group dynamics, such as the geographic distance between partners and the burden of staff/practitioners having to facilitate team-building exercises. In addition, this study found that a group dynamics-based tool in an online intervention may provide a more engaging social environment for participants to interact in than those of a standard social support app. Further study is needed to determine whether incorporating group dynamics-based principles into PA interventions of longer duration can impact cohesion and PA in real-world settings.

## Abbreviations

ICC: intraclass correlation coefficient
MVPA: moderate-to-vigorous physical activity
PA: physical activity
PAGEQ: Physical Activity Group Environment Questionnaire
RPE: ratings of perceived exertion
